# Differences in beginner and expert neurointerventionalists” heart rate variability during simulated neuroangiographies

**DOI:** 10.1177/15910199221128439

**Published:** 2022-09-19

**Authors:** Dominik Hinzmann, Maximilian Singer, Valerie Schmelter, Kornelia Kreiser, Kim Gehling, Lea Ströber, Jan S Kirschke, Christian M Schulz, Frederick Schneider

**Affiliations:** 1 TUM School of Medicine, Department of Anesthesiology and Intensive Care, Technical University of Munich, München, Germany; 2 Department of Opthalmology, Ludwig-Maximilians-University, LMU Klinikum, München, Germany; 3 TUM School of Medicine, Department of Diagnostic and Interventional Neuroradiology, Technical University of Munich, München, Germany; 4 RKU - University and Rehabilitative Hospitals Ulm, Ulm, Germany; 5 Department of Diagnostic and Interventional Radiology, Heidelberg University Hospital, Heidelberg, Germany; 6 Department of Urology and Children’s Urology, RoMed Klinikum Rosenheim, Rosenheim, Germany

**Keywords:** Simulation, heart rate variability, performance, neuroradiology, knowledge

## Abstract

**Background:**

Likewise work experience, heart rate variability (HRV) has repeatedly been correlated with improved performance under real life and simulator conditions. Using HRV as a correlate of workload, it is meaningful to assess the impact of work experience. To understand the impact of work experience on HRV metrics, we examined differences in HRV among experts and beginners during simulated endovascular neuroradiological procedures.

**Methods:**

Six inexperienced radiologists (beginners) and five experts in neurological endovascular intervention each performed 10 diagnostic angiographies on a Vascular Interventional System Trainer (VIST) simulator (Mentice AB, SW). Beyond total time, fluoroscopy time, and amount of contrast medium used, heart rate variability and the NASA-task load index were gathered as correlates of workload. The t-Test for independent samples as well as Mann-Whitney-U tests were applied for group-wise comparison between beginners and experts. Multivariate regression was used to assess the influence of age and expert status.

**Results:**

Ten participants completed all scenarios; one participant only completed the first five scenarios. Accordingly, 105 simulations were analyzed (beginners N = 60; experts N = 45, respectively). The heart rate variability of experts and beginners significantly differed in three time domain HRV metrics (decreased RMSSD, NN50, pNN50 in experts; all p < 0.05) as well as with respect to its distribution in the frequency spectrum (LF/HF ratio; p < 0.001, increased high frequency components in experts).

**Conclusions:**

The HRV of beginners and expert neurointerventionalists significantly differed during simulated endovascular neuroradiological procedures. Experts presented decreased HRV, this could be a cardiovascular surrogate to the effort the subjects expend on their performance. It is in line with previous studies on vagal influences on the heart and cognitive-executive performance.

## Background

Due to growing demand for the interventional treatment of strokes, a sufficient availability of interventional neuroradiologists is crucial to offer rapid treatment.^1–3^ Simulations of neuroangiographic procedures are a simple and effective method to train interventional neuroradiologists at a high level.^4–6^

Especially in a medical environment, work experience has repeatedly been correlated with improved performance: More experienced anesthetists, for example, were anticipated to be faster and more efficient while providing general anesthesia.^7^ Likewise, in a simulation by Schulz and colleagues, more experienced anesthetists spent more time on manual tasks during simulated-critical incidents; they assume this might be a correlate of a quicker reaction under challenging circumstances and thus experts” performance.^8^ Accordingly, in their meta-analysis of work experience, Quiñones and Ford revealed that there is a strong interaction between work experience and performance that is independent from the measure used to quantify work experience.^9^ From the time when it has first been used as a performance marker in combat flight simulation, heart rate variability (HRV) was used to evaluate workload and performance in various medical and non-medical simulation environments.^8,10–13^

According to the situational awareness framework, an explanation for the impact of work experience on performance is that more experienced subjects develop sophisticated mental models and automatisms for information-procession, and therefore have additional cognitive spare capacity for the challenges of an unknown situation.^14–16^ According to the neurovisceral integration model proposed by Thayer and colleagues, these mental models and spare capacities might be represented by vagally-mediated HRV metrics since they are correlates of cognitive performance, and especially index prefrontal neuronal activity.^17^

Such vagally-mediated HRV metrics are predominately found among the frequency-domain HRV metrics; i.e., these measures of HRV that are calculated based on the power spectral density of the signal.^17,18^ Moreover, Beh and colleagues could also link time-domain HRV metrics (i.e., HRV measures directly calculated from the variance in RR-intervals, in their case inter-beat-intervals, IBIs) with performance.^19^ Their study revealed that people with melior performance showed lower HRV; this could be a cardiovascular reaction to the effort the subjects expend on their performance.^19^ When taking stressful situations into account, a greater cardiac vagal tone under resting conditions has been associated with superior executive performance under threat and non-threat conditions (Hansen, Johnsen, & Thayer, 2003, 2009; Thayer *et al*., 2009). In line, Schneider and colleagues found HRV metrics a promising tool to discriminate workload levels during anesthesia and pre-hospital emergency care.^12,13^

However, the impact of work experience on HRV metrics during medical procedures has not yet been systematically examined. Thus, the exploratory aim of this study was to examine differences in HRV metrics among experts and beginners during simulated endovascular neuroradiologic procedures. Therefore, HRV of expert neurointerventionalists and beginners during ten simulated cerebral angiographies was analyzed and compared. We hypothesized that HRV metrics, particularly these associated with vagal-mediation, as well as perceived task load (measured by the NASA task load index) would be associated with the work experience of neuroradiologists.

## Methods

### Study setting

Ten participants were recruited from the radiology and neuroradiology departments at a German university hospital. According to their experience in neuroradiology, the groups were divided into a beginner group (N = 6) with little or no interventional neuroradiologic experience and an expert group (N = 5) with advanced experience in cerebral angiography. Participants in the beginner group had each performed less than 30 cerebral angiographies prior to their participation in the study, while the expert group consisted of five participants with an experience of 100 or more cerebral angiographies undertaken per physician.^20^

All participants performed ten cases that each consisted of a complete cerebral angiography in one patient. The cases were classified into five categories where the most difficult level required detailed knowledge of cerebral vessel anatomy as well as advanced catheter maneuvers.^20^ A technical introduction with an exercise case that was not part of the study preceded the study to ensure that all participants were familiar with the simulator environment. Simulation sessions were limited to 2h in order to avoid fatigue effects. During the cases the HRV of all participants was recorded using a chest belt (see below for details). Additionally, workload was evaluated after each case using the task load index of the National Aeronautics and Space Agency (NASA-tlx). Informed consent has been obtained from all participants prior to participation and the local ethics committee approved the study. In accordance with prior studies, no restrictions concerning food intake, activity, sleep or medication were imposed before and during the participation.^12,13^

### Simulator setting

The high-fidelity vascular intervention system trainer VIST C (Mentice AB, Gothenburg, Sweden) was used to simulate the neuroangiographies. It was integrated in the VIST LAB (Mentice AB, Gothenburg, Sweden), a stationary unit that resembles a vascular intervention workspace including a patient silhouette and multiple screens to depicture x-rays, additional information and roadmaps. An insertion sheath at the height of the simulated patients groin serves as an access point for various interventional devices and detects movements like pushing, pulling and rotation of the introduced wires and catheters via three sensors. Catheter movements are transferred into a virtual patient anatomy that is displayed on the screens in real-time. Furthermore, a footswitch with two pedals as well as a panel with several options (control of the virtual c-arms, shutter, zoom, etc.) are part of the VIST LAB angiography simulator. Further details about the simulator setting as well as the presented scenarios can be found, elsewhere.^20^

### Computation of HRV metrics

For the computation of HRV metrics a chest belt (Zephyr™ Bio Harness 3, Medtronic, Dublin, Ireland) was used to gather continuous electrocardiographs (ECG) of the participants during the simulator sessions. Hereafter, the raw ECGs were processed using the MATLAB®-based software tool ARTiiFACT 2.2 (Biosignal Analysis and Medical Imaging Group, Department of Applied Physics, University of Eastern Finland, Kuopio, Finland).^21^ A high-pass filter of 10 Hertz (Hz) and a global threshold of 50µV were used to warrant optimized R-peak detection. In accordance with the guidelines proposed by the Task Force of the European Society of Cardiology and the North American Society of Pacing and Electrophysiology the detected peaks were visually screened for accuracy.^18^ Inaccurately detected R peaks were manually adjusted. Again, the ARTiiFACT software was used to calculate the inter-beat-intervals (IBIs) from the R-peak data, the built-in artefact detection algorithm (based on work by Berntson and and colleagues) revised incorrect IBIs using cubic spline interpolation.^21,22^ Finally, the artefact corrected IBI were used for the computation of time and frequency domain HRV metrics, again using the freeware ARTiiFACT.^21^

In general, HRV metrics are classified in two underlying categories: First, the HRV metrics from the time-domain. These are estimated from the statistical analysis of RR intervals. Second, frequency-domain HRV metrics computed from the analysis of the frequency spectrum calculated from RR interval series via Welch’s periodogram.

From the time domain, mean heart rate (mean HR) and the related HRV metrics, i.e., the mean value of RR intervals (mean RR, ms), the standard deviation of normal-to-normal RR intervals (SDNN, equivalent of short-term high frequency variations), the root mean square of successive differences (RMSSD, estimate of short-term components of HRV), the number of successive intervals differing more than 50 ms (NN50), and the corresponding relative amount (pNN50, both estimates of short-term components, respectively) were included.^23^ Units as well as a more detailed description of the time domain HRV metrics are presented in Supplementary Table 1.

Beyond time-domain HRV metrics, frequency-domain methods analyze power spectral densities in order to describe how variance distributes as a function of frequency.^18^ Frequencies were divided in three frequency bands 1) very low frequencies (VLF) ranging from 0 to 0.04 Hz, 2) low frequencies (LF) from 0.04 to 0.15 Hz, and 3) high frequencies from 0.15 to 0.4 Hz.^18^ From the frequency-domain HRV metrics, absolute and relative powers (of VLF, LF, and HF), normalized powers of LF and HF (n.u., relative power of LF and HF after subtraction of the VLF from the total power), and the LF/HF power ratio were included. The software calculates the corresponding powers as the integral of the spectrum estimates over the frequency bands.^23^ See the supplementary information for a detailed table on computation, units and calculations of frequency based HRV metrics.

### Statistical analysis

Differences in heart rate variability and NASA-tlx between experts and beginners were evaluated using the t-test for independent samples (metric variables) and the Mann-Whitney-U test for group comparisons (non-metric variables). For metric variables with unequal variances, Welch’s correction was applied. To control for the influence of age on HRV metrics, we calculated multivariate regression analysis to evaluate the influence of age and expert status on the HRV metrics. Metric data are presented as mean (± standard deviation, SD). SPSS Statistics 27.0 (IBM Corporation, Armonk, NY, USA) was used for statistical analysis. Statistical significance was set at a p-value of <0.05. To account for multiple testing within the same dataset the Benjamini-Hochberg procedure (BH-stepup procedure) was applied, post-hoc.^24^

## Results

### Demographic data

11 physicians participated in the study. Of them, five were allocated in the expert group due to their advanced neuroangiographic experience, while six subjects were grouped in the beginner group. These six participants consisted of four unexperienced neuroradiology residents and two radiological residents with only peripheral angiographic experience. One participant of the expert group only performed the first five simulations. See Table 1 for further demographic data like age, experience and sex.

**Table 1.** Demographic data of participants in the beginner and expert group.

	*BEGINNERS (n* *=* *6)*	*EXPERTS (n* *=* *5)*
Age (mean [range]; yrs.)	31.86 [27-37]	42.25 [40–47]
Sex (f/m; n)	(3/3)	(0/5)
Experience in cerebral angiographies (mean [range]; yrs.)	0 [0–3]	4 [2–10]

Table 1 presents the core demographic data of participants in the beginner and expert group. Experts performed more than 100 cerebral angiographies each. Abbreviations: yrs, years; f, female; m, male.

### Heart rate variability differences between experts and beginners in neuroradiology

Mean heart rate (mean HR) did not differ among beginners and experts during cerebral angiographies, while three parameters of time domain HRV and six parameters of frequency domain HRV were significantly different between the groups (see Table 2 for details). Except for RMSSD (a time-domain estimate of short-term HRV) these results remained consistent after the Benjamini-Hochberg procedure was computed. Perceived workload in terms of mental, physical and temporal demand values gathered from the NASA-tlx was not different between the groups. Table 2 presents the means (± standard deviation, SD) of the respective time and frequency domain HRV metrics. Medians and interquartile-ranges for NASA-tlx scores (±SD) for mental, physical and temporal demands are presented in Table 3, respectively.

**Table 2.** Differences in heart rate variability metrics between beginners and experts in cerebral angiography.

	BEGINNERS		EXPERTS			
	Mean	SD	Mean	SD	P	BH-p
HRV METRICS						
*Time domain*	* *	* *	* *	* *	* *	* *
Mean RR	757.0	126.3	770.5	93.7	0.548	0.613
Median RR	755.8	129.7	768.6	98.2	0.582	0.613
Mean HR	81.5	13.9	78.9	8.8	0.253	0.370
SDNN	56.8	17.7	57.6	19.7	0.838	0.838
RMSSD	31.4	13.2	25.8	13.9	0.039*	0.082
NN50	41.4	36.9	22.8	30.7	0.007*	0.022*
pNN50	10.2	8.4	6.0	8.0	0.012*	0.033*
*Frequency domain*	* *	* *	* *	* *	* *	* *
VLF%	38.4	16.2	47.3	15.9	0.006*	0.022*
LF%	50.0	15.5	46.0	16.2	0.205	0.337
HF%	11.6	4.9	6.7	4.1	<0.001**	0.001*
LF/HF	5.2	3.2	10.2	7.6	<0.001**	0.001*
LF n.u.	80.3	8.8	86.0	8.9	0.001*	0.005*
HF n.u.	19.7	8.8	14.0	8.9	0.001*	0.005*
VLF abs	1360.0	1655.8	1762.1	1573.8	0.213	0.338
LF abs	1762.6	1509.0	1533.8	1166.1	0.400	0.532
HF abs	400.7	417.2	232.9	257.3	0.019*	0.045*

Table 2 presents the means (± standard deviation, SD) of the respective time and frequency domain HRV metrics and the results from the comparison between the groups in before (p) and after (BH-p) correction for repeated testing within the same dataset. Abbreviations: *, p < 0.005, **, p < 0.001; SD, standard deviation; HRV, heart rate variability; RR, distance between two subsequent R peaks, SDNN, Standard deviation of normal-to-normal RR- intervals; RMSSD, Square root of the mean squared differences between successive RR-intervals; NN50, Number of successive RR-interval pairs that differ more than 50 ms; pNN50, NN50 divided by the total number of RR-intervals; VLF, Very low frequency (0–0.04 Hz); LF, Low frequency (0.04–0.15 Hz); HF, High frequency (0.15–0.4 Hz), VLF, LF and HF powers [%],Relative powers of VLF, LF and HF bands; LF/HF, ratio between HF and LF band power; LF and HF [n.u.], Powers of LF and HF bands in normalized units; VLF, LF, and HF abs., absolute powers of VLF, LF, and HF bands.

**Table 3.** Subjective workload differences between beginners and experts in cerebral angiography.

	BEGINNERS		EXPERTS			
	Median	[IQR]	Median	[IQR]	P	BH-p
NASA-TLX						
Mental	11.5	[6.75–16]	9	[5.5–13.5]	0.198	0.338
Physical	4	[4–6]	4	[3–7]	0.420	0.532
Temporal	6.5	[4.75–11.25]	8	[4.5–11.5]	0.492	0.584

Table 3 presents the median [interquartile range] of the respective NASA task load index subcategories from the comparison between the groups in before (p) and after (BH-p) correction for repeated testing within the same dataset. Abbreviations: NASA-TLX, NASA task load index.

### Influence of expert status and age on HRV metrics

While, from the time domain, mean RR, median RR and mean HR were significantly influenced by both, expert status and participants” age (p ≤ 0.001 for all) in the multivariate regression analysis (Table 4), pNN50 was only connected with expert status. Likewise, five frequency domain measures were influenced by expert status but not by the age of the participants. See Table 4 for further details.

**Table 4.** Influence of expert status and age on HRV metrics.

		EXPERT		AGE	
	*model significance (p)*	*T*	*p*	*T*	*p*
HRV METRICS					
*Time Domain*					
Mean RR	<0.001**	−4.36	<0.001**	6,12	<0.001**
Median RR	<0.001**	−4.22	<0.001**	5.80	<0.001**
Mean HR	<0.001**	3.34	0.001*	−5.21	<0.001**
SDNN	0.733	−0.47	0.638	0,76	0.447
RMSSD	0.093	−1.85	0.067	0.71	0.480
NN50	0.027*	−1.79	0.076	0.12	0.904
pNN50	0.020*	−2.56	0.012*	1.24	0.218
*Frequency Domain*					
VLF %	0.021*	1.38	0.172	0.46	0.649
LF %	0.400	−0.41	0.683	−0.48	0.630
HF %	<0.001**	−3.41	0.001*	0.07	0.944
LF/HF	<0.001**	2.83	0.006*	−0.03	0.978
LF n.u.	0.005*	2.67	0.009*	−0.81	0.418
HF n.u.	0.005*	−2.67	0.009*	0.81	0.418
VLF abs.	0.289	0.02	0.987	0.97	0.334
LF abs.	0.699	−0.60	0.548	0.10	0.918
HF abs.	0.062	−1.20	0.734	−0.35	0.729

Table 4 presents the influence and significance of linear multivariate regression analysis comparing the influence of expert status and age on HRV metrics. Abbreviations: Expert, comparison between experts and beginners; T, regression coefficient of the respective t-tests; p. significance; *, p < 0.005, **, p < 0.001; SD, standard deviation; HRV, heart rate variability; RR, distance between two subsequent R peaks, SDNN, Standard deviation of normal-to-normal RR- intervals; RMSSD, Square root of the mean squared differences between successive RR-intervals; NN50, Number of successive RR-interval pairs that differ more than 50 ms; pNN50, NN50 divided by the total number of RR-intervals; VLF, Very low frequency (0–0.04 Hz); LF, Low frequency (0.04–0.15 Hz); HF, High frequency (0.15–0.4 Hz), VLF, LF and HF powers [%],Relative powers of VLF, LF and HF bands; LF/HF, ratio between HF and LF band power; LF and HF [n.u.], Powers of LF and HF bands in normalized units; VLF, LF, and HF abs., absolute powers of VLF, LF, and HF bands

## Discussion

The aim of the present analysis was to examine differences in HRV metrics among experts and beginners during simulated endovascular neuroradiologic procedures. HRV significantly differed between expert neuroradiologists and beginners in three time-domain and several frequency-domain HRV metrics. Except for one time domain estimate of short-term HRV (RMSSD), these differences remained significant after correction for multiple testing. Furthermore, most of the frequency domain metrics were significantly influenced by expert status, while they were not influenced by age. Thus, we assume that differences in HRV measures are associated with neuroradiologists” experience in cerebral angiography.

From the time-domain of HRV metrics mostly estimates of short-term variability (RMSSD, p = 0.039, NN50, p = 0.007, and pNN50, p = 0.012, respectively), did significantly differ between experts and beginners. Since work experience is known to interact with performance in medicine^7,14^ and experts performed significantly better in these simulated cases (results described in depth, elsewhere),^20^ these HRV metrics could be considered as correlates of performance. Predominantly for RMSSD, this is in line with findings from other studies on the topic.^25,26^ Yet, the results were not consistent after correction for multiple testing.

In their neurovisceral integration model Thayer and colleagues precise these interactions between HRV metrics, parasympathetic influences on the sinoatrial pacemaker and cognitive-executive performance.^17^ They mainly focus on HRV metrics from the frequency-domain. In the present analysis, experts and beginners differed in six of nine frequency-domain HRV metrics (Table 2). Repetitively, the high frequency (HF) component was higher in beginners than experts, and HF components were only influenced by experience and not age in the multivariate regression analysis (Table 4). This is an interesting finding, as HF components of HRV are said to be chiefly influenced by vagal stimulation, and thus higher shares of HF signals are presumed to be correlates of a vagal body reaction.^17,18^ Thus, elevated HF components are a reaction one would – during a challenging task – rather expect from experts than beginners. Nonetheless, high shares of HF components have been found in inferior groups, before.^16^ Elliot and Colleagues linked decreased HF activity to worse IQ performance.^27^ For our sample, however, decreased HF components (and thus, according to Elliot and colleagues diminished IQ performance) might simply equal a more automated course of action within the more experienced participants. This is in line with results published by Helen Beh: she proposed that performance reached through achievement motivation is reflected in HRV metrics.^19^ With regards to subjective workload analysis via the NASA task load index, beginners rate the mental demands at 11.5 [6.75–16] (of 20; median [interquartile range]), while experts median mental demands are 9 [5.5–13.5] (median [interquartile range]; Table 3).

To date, HRV metrics have been extensively studied insimulator environments from combat flight and aviation ^28–30^ to medical simulation.^16^ Yet, differences between experts and beginners have only seldom been investigated. Conversely, these differences are majorly important for a potential use of HRV metrics to prevent harmful events: When there is need to detect the risk of work overload, it is meaningful to define a threshold between experts and beginners. Interestingly, the NASA task load index did not differ between both groups in terms of mental, temporal and physical demands (Table 2). Yet, beginners rate mental demands at 11.5 [6.75–16] (median [interquartile range]; vs. 9 [5.5–13.5] in experts), whereas experts – used to the typical production pressure of their work environment – experience the simulator settings rather timely demanding (8 [4.5–11.5], median [interquartile range]; vs. 6.5 [4.75–11.75] in beginners). Nonetheless, it has to be kept in mind that the simulation did neither include real patients nor real radiation, and thus workload levels might have been considerably lower than under real-life circumstances.

In a clinical environment, high levels of workload and stress can impair patient safety. Hence, it is of particular interest to measure workload and prevent work-overload. Physiological workload correlates such as HRV have proven useful to differentiate workload stages in the operation theatre as well as emergency medicine.^12,13^ Future research needs to concentrate on the real-time analysis of heart rate variability and the definition of individual thresholds allowing a remote monitoring to trigger expert support and impede adverse events caused by work- overload. Yet, it is necessary to identify threshold between beginners and advanced practitioners, to minimize “false alarms” during challenging situations that could easily be solved by advanced practitioners without expert support.

Notwithstanding, there are some limitations to consider. First, the number of participants for this study was low and experience level within the beginner group was rather heterogeneous. However, our findings are supported by larger studies.^12,16^ And we thus do not think that the sample size effected our results. Second, in line with previous work^12,16^ and the neurovisceral integration model,^17^ we did not account for age corrections. However, the multivariate regression model showed that the differences we observed were significantly associated with experience and not age. Likewise, with a range from 27 to 47 the age group for the present study was rather homogeneous; especially, since age-related effects on HRV metrics are mostly seen in the very old and the very young.^31,32^ Furthermore, we did not record baseline values for HRV, since we assumed that the external workload exerted by the cerebral angiography tasks was comparable for beginners and experts. However, one can never completely rule out that there were external factors (e.g., daytime, previous experiences throughout the day of the study, motivation to participate) that made some participants perform better than others. These external factors might influence HRV metrics. Beyond, in a population consisting of healthy doctors and medical students, we assumed that general health status and fitness levels are comparable for both groups. Furthermore, although the participants did not report differences in task demands (Table 3), we can only assume that differences in vagal activities did not result from some participants giving up and relaxing due to too difficult tasks. However, in the absence of real patients and real radiation, the transferability of our results to real-life circumstances is limited, since workload levels could be considerable higher once real patients are involved. Alike, the absence of restrictions concerning medication or food intake might influence our results and impair their comparability with other studies. Yet, this practice is common in medical research on HRV ^26,33^ and resulted from the study setting.

In brief, this study unveils the capabilities of HRV metrics, mainly from the frequency-domain, to discriminate experts and beginners during simulated medical procedures. This is in line with previous studies and reflects interactions of vagal influences on the heart and cognitive-executive performance.

## Authors' contributions

DH drafted the text and wrote the final version of the manuscript. MS analyzed the data and helped writing the manuscript. KK conducted and designed the study. KG and LS collected the data. JSK and CMS contributed with important analysis tools and supervised the project. VS helped with statistical analysis. FS performed the statistical analysis supervised the study. All authors discussed the results and approve the final version the manuscript.

## Availability of data

The data are available from the corresponding author upon reasonable request

## Consent to participate

Informed consent has been obtained from all participants prior to participation.

## Consent for publication

All authors discussed the results and approve the final version the manuscript.

## Supplemental Material

sj-docx-1-ine-10.1177_15910199221128439 - Supplemental material for Differences in beginner and expert neurointerventionalists” heart rate variability during simulated neuroangiographies Supplemental material, sj-docx-1-ine-10.1177_15910199221128439 for Differences in beginner and expert neurointerventionalists” heart rate variability during simulated neuroangiographies by Dominik Hinzmann, Maximilian Singer, Valerie Schmelter, Kornelia Kreiser, Kim Gehling, Lea Ströber, Jan S Kirschke, Christian M Schulz and Frederick Schneider in Interventional Neuroradiology

## List of abbreviations

Abbreviation: Definition
ECG: Electrocardiogram
HF: High frequency (0.15 to 0.4Hz)
HRV: Heart rate variability
Hz: Hertz [unit]
IBI: Inter-Beat-Interval – Interval between two successive R-peaks in the ECG
IQR: Interquartile range
LF: Low frequency (0.04 to 0.15Hz)
NASA: National Aeronautics and Space Agency
NN50: Absolute number of successive RR-interval pairs that differ more than 50 ms
pNN50: Relative number of successive RR-interval pairs that differ more than 50 ms
RMSSD: Square root of the mean squared differences between successive RR-intervals
RR: Interval between two successive R-peaks in the ECG
SD: Standard deviation
SDNN: Standard deviation of normal-to-normal RR-interval
VIST: Vascular Interventional System Trainer
Vlf: Very Low Frequency (0 To 0.04hz)
