# Supplementary material for: Differences in beginner and expert neurointerventionalists” heart rate variability during simulated neuroangiographies
Source: Interv Neuroradiol. 2022 Sep 19;30(2):195–201. doi: 10.1177/15910199221128439 (PMC11095349; doi:10.1177/15910199221128439)
Supplement: sj-docx-1-ine-10.1177_15910199221128439 - Supplemental material for Differences in beginner and expert neurointerventionalists” heart rate variability during simulated neuroangiographies [file sj-docx-1-ine-10.1177_15910199221128439.docx]

**Supplementary Table** **1** – Detailed description of heart rate variability metrics

| Parameter | Units | Description |
| --- | --- | --- |
| Time Domain |  |  |
| Mean RR | [ms] | The mean of all RR-intervals |
| SDNN | [ms] | Standard deviation of normal-to-normal RR-intervals |
| Mean HR | [1/min] | The mean heart rate |
| STDHR | [1/min] | Standard deviation of instantaneous heart rate values |
| RMSSD | [ms] | Square root of the mean squared differences between successive RR-intervals |
| NN50 | [count] | Number of successive RR-interval pairs that differ more than 50 ms |
| pNN50 | [%] | NN50 divided by the total number of RR-intervals |
| Frequency Domain | *All frequency domain heart rate variability metrics are delivered based on spectrum estimates computed according to Welch’s periodogram.* | |
| VLF, LF, and HF powers | [ms^2^] | Absolute powers of VLF, LF, and HF bands |
| VLF, LF, and HF powers | [%] | Relative powers of VLF, LF, and HF bands  VLF [%] = VLF [ms^2^]/total power [ms^2^] x 100 %  LF [%] = LF [ms^2^]/total power [ms^2^] x 100 %  HF [%] = HF [ms^2^]/total power [ms^2^] x 100 % |
| LF and HF powers | [n.u.] | Powers of LF and HF bands in normalized units  LF [n.u.] = LF [ms^2^]/(total power [ms^2^] - VLF [ms^2^])  HF [n.u.] = HF [ms^2^]/(total power [ms^2^] - VLF [ms^2^]) |
| LF/HF | - | Ratio between LF and HF band powers |

Overview on the heart rate variability metrics computed for analysis. Abbreviations: ms, miliseconds; min, minutes; VLF, very low frequencies; LF, low frequencies; HF, high frequencies. Modified version of the table delivered in Tarvainen, Niskanen et al. 2014.
